# Retinal Microvasculopathy Is Common in HIV/AIDS Patients: A Cross-Sectional Study at the Cape Coast Teaching Hospital, Ghana

**DOI:** 10.1155/2016/8614095

**Published:** 2016-12-29

**Authors:** Emmanuel Kwasi Abu, Samuel Abokyi, Dorcas Obiri-Yeboah, Richard Kobina Dadzie Ephraim, Daniel Afedo, Lawrence Duah Agyeman, Samuel Bert Boadi-Kusi

**Affiliations:** ^1^Department of Optometry, School of Allied Health Sciences, University of Cape Coast, Cape Coast, Ghana; ^2^Department of Microbiology and Immunology, School of Medical Sciences, University of Cape Coast, Cape Coast, Ghana; ^3^Department of Medical Laboratory Science, School of Allied Health Sciences, University of Cape Coast, Cape Coast, Ghana

## Abstract

*Purpose*. The purpose of this study was to evaluate the ocular disorders in HIV positive patients attending the Cape Coast Teaching Hospital, Ghana.* Methods*. A cross-sectional study using systematic random sampling was conducted on 295 HIV positive patients. Data collection consisted of semistructured questionnaires, laboratory investigation, medical profile, and ophthalmic examination. Statistical association tests including *χ*^2^, independent *t*-test, and ANOVA were done. A *p* value ≤ 0.05 was considered statistically significant.* Results*. Of the 295 participants, 205 (69.5%) were on antiretroviral therapy while 90 (30.3%) were not on therapy. Majority of the participants (162, 54.9%) were in clinical stage two, followed by stages three (68, 23.1%), one (62, 21%), and four (3, 1%), respectively. The overall prevalence of ocular disorders was 5.8%. The most common HIV related ocular disorder was HIV retinal microvasculopathy (58.8%), followed by herpes zoster ophthalmicus and* Toxoplasma* retinochoroiditis, both representing 11.8% of ocular disorders seen. Cytomegalovirus retinitis, Bell's palsy, and optic neuritis were the least common (5.9%). CMV retinitis recorded the highest viral load of 1,474,676 copies/mL and mean CD4 count of 136 cells/mm^3^. The mean CD4 count for participants with HIV related ocular disorders was significantly lower compared to participants without disorders (*t* = 2.5, *p* = 0.012). Participants with ocular disorders also recorded significantly higher mean viral loads than those who did not have ocular disorders (*t* = 2.8, *p* = 0.006).* Conclusion*. Lower CD4 counts and high viral load copies were associated with the manifestation of HIV related ocular disorders.

## 1. Background

Acquired immunodeficiency syndrome (AIDS) is caused by the human immunodeficiency virus (HIV) which affects all body organs either directly or by opportunistic infections, and the eye is not spared. AIDS indicates advanced HIV disease in which progressive failure of the immune system allows life-threatening opportunistic infections and cancers to thrive [1]. Opportunistic disease presentation differs according to different world regions as a result of different local prevalence of opportunistic agents. The introduction of highly active antiretroviral therapy also seems to present another range of toxic pathological damage [2]. Since the first ever case of HIV was diagnosed in the US in 1981, it has become a major public health concern in all economies [3]. In 2014, there were approximately 36.9 million people living with HIV (2.6 million being children) across the world with about 70% of the cases living in sub-Saharan Africa. It is also reported that 2 million people became newly infected with HIV globally in 2014 and 1.2 million people lost their lives to AIDS-related illness in the same year [4]. The first case of HIV in Ghana was diagnosed in March 1986 and though the general prevalence and incidence rates seem to be declining, they are still far from acceptable. The national HIV prevalence is estimated at 1.47% [5]. Holland et al. were the first to report ocular manifestations of HIV/AIDS and, thereafter, ocular manifestations have been reported in up to 70% of individuals infected with HIV [6]. Ocular manifestations reflect systemic diseases and may be the first sign of disseminated infection in an otherwise asymptomatic patient [3, 7]. It is estimated that about 70 to 80% of adult HIV/AIDS patients will experience ocular complication at some point of their illness. HIV related ocular disorders result either from the direct destruction of the eye by the virus or from the actions of opportunistic infections. Such ocular disorders include cytomegalovirus (CMV) retinitis, molluscum contagiosum, HIV retinal microvasculopathy (HIV RM), keratoconjunctivitis sicca, herpes zoster ophthalmicus (HZO), Kaposi sarcoma, and neuroophthalmic lesions [7, 8]. CMV retinitis which is associated with extreme immune suppression and high viral load is the main cause of blindness in that population [8, 9]. CMV retinitis, however, seems to be rare in African patients. The patterns of ocular diseases in HIV patients also differ across different world regions as a result of possible early mortality of the patients in developing countries, differences in HIV subtype, race, influence of comorbidity, and cultural and social barriers to testing and treatment [7, 10].

There is a huge dearth of information in Ghana on ocular related findings in HIV patients. Reports from other societies have always considered the associations between HIV related ocular manifestations and CD4 counts but not viral loads. The purpose of this study was to evaluate the associations between presenting ocular disorders and CD4 counts/viral load copies in HIV positive patients attending the Cape Coast Teaching Hospital, Ghana.

## 2. Methods

### 2.1. Study Site

The study was carried out at the Antiretroviral Therapy (ART) Specialist Clinic of the Cape Coast Teaching Hospital in Ghana. The Cape Coast Teaching Hospital, one of the five teaching hospitals in Ghana, provides health services to people in the central and western regions and parts of Ashanti and eastern regions. It also has the mandate of training doctors and other medical staff.

### 2.2. Study Design and Participants

The study employed a descriptive cross-sectional design. Sample size was determined based on the expression *N* = *Z*^2^(1 − *p*)(*p*)/*b*^2^ [11], where *N* is the minimum sample size, *z* is the standard normal deviation, usually set at 1.96 which corresponds to the 95% confidence interval, *p* is the proportion in the target population estimated to have the infection (1.4% for the central region), and *b* is the degree of accuracy desired, usually set at 5%. Consequently, a sample size of 30 was calculated. This was adjusted to 200 participants to account for attrition rate and inefficiencies associated with the sampling method. A systematic sampling method was used to recruit clinically diagnosed HIV patients, who attended the ART Specialist Clinic between January and May 2016. Based on the average daily attendance at the clinic and a determined sampling interval of three (3), the first participant was chosen by randomly selecting a number between one and three. Every third patient from the first participant was then recruited into the study. In all, 320 patients were examined within the period. Out of them, 295 patients had complete medical data and they were included in the analysis. Patients who had comorbidities such as diabetes, hypertension, and sickle cell disease were excluded from the study.

### 2.3. Ethical Considerations

The study was conducted in accordance with the Helsinki Declaration on Research Regarding Human Subjects. Ethical clearance (ID number UCCIRB/CHAS/2015/090) was obtained from the Institutional Review Board of the University of Cape Coast. The rationale of the study was comprehensively explained to all participants after which consent forms were issued to them to sign, including local language versions which were read out to illiterate subjects who consented with a thumbprint prior to investigation. For participants below 18 years of age whose assent was sought, their parents/guardians signed the consent forms on their behalf. The participants were assured of confidentiality and safety at all times. They were also reminded of the voluntary nature of the study which granted them the right to withdraw at any point.

### 2.4. Data Collection

The method of data collection consisted of semistructured questionnaires, laboratory investigation, medical profiles, and ophthalmic examination. Every consenting patient was assigned a specific study code. A trained investigator interviewed and filled the questionnaires for all the participants. Information obtained from the questionnaires included age, gender, occupation, religion, marital status, level of education, and duration of infection from the time of diagnosis. The patient's HIV related medical information such as the World Health Organization (WHO) clinical stage of the disease, whether the participant was on ART or not, and duration of ART usage was recorded.

### 2.5. Blood Sample Collection and Analysis

About 3 mL of venous blood was collected from each participant and HIV biomarkers CD4 counts and viral loads were determined. Two CD4 counts were obtained for this study: nadir CD4 counts (the lowest CD4 count ever recorded) and the current CD4 counts (CD4 counts at the time of the study). It was important to evaluate both the nadir and the current CD4 counts in this study to appreciate the effect of antiretroviral therapy on CD4 and to ascertain the stage at which some HIV related ocular disorders actually occurred.

### 2.6. Ophthalmic Examination

Ophthalmic examination was conducted by experienced eye care personnel. Presenting Distance Visual Acuity (PDVA) measurements followed by slit lamp biomicroscopy and dilated funduscopy were performed on all participants. Pinhole acuity was performed to rule out refractive error as the cause of reduced vision. Dilated fundus examination was performed with 2.5% phenylephrine ophthalmic solution. Two drops of the phenylephrine were administered at an interval of 5 minutes followed by one hour of waiting to ensure maximum papillary dilation. Ophthalmoscopy was then performed on each participant. Ophthalmic examination forms were correspondingly labeled for each participant. Categories of visual impairment (VI) were determined based on the International Classification of Diseases, where “low vision” is defined as presenting visual acuity < 6/18 in the better eye [12].

### 2.7. Statistical Analysis

The data collected were entered into SPSS for Windows, version 21.0.1. The analysis involved the use of frequency distribution tables. Chi-square (*χ*^2^) test was used to determine the associations between categorical variables. Fisher's exact test was used where counts were less than 5. Independent *t*-test and analysis of variance (ANOVA) were performed to compare the mean values of HIV biomarkers (CD4 counts and viral loads) between participants who had HIV related visual disorders and those who did not. A *p* value ≤ 0.05 was considered statistically significant.

## 3. Results

A total of 320 patients were examined within the period, out of whom 295 had complete medical data and were included in the analysis. They comprised 78 (26.4%) males and 217 (73.6%) females. Their ages ranged from 10 to 83 years with mean age of 45.3 years (SD: ±11.6). According to the WHO criteria on HIV classification, majority of the participants (162, 54.9%) were in clinical stage two, followed by stages three, one, and four that had 68 (23.1%), 62 (21%), and 3 (1%) participants, respectively. Two hundred and five (69.5%) of the participants were on antiretroviral therapy while the remaining 90 (30.5%) were not on therapy. The mean age of participants on ART was 45.9 (SD: ±11.64) years while those not on ART had a mean age of 43.8 (SD: ±11.46) years. There was no statistically significant difference between the mean ages of participants on ART and those without therapy (*t* = 1.4, *p* = 0.14). The mean current CD4 count of participants on ART was lower (786.5 cells/mm^3^) than of participants not on ART (863.8 cells/mm^3^). This difference was, however, not significant (*t* = 1.3, *p* = 0.20). Similarly, there was no significant difference between the mean viral load counts for participants on ART and those not on ART (*t* = 1.1, *p* = 0.28).

Majority of the participants (150, 50.8%) had normal vision of VA 6/6. One hundred and sixteen (39.3%) had mild visual impairment (VA 6/9–6/18). Twenty-nine (9.8%) of the participants had low vision (VA < 6/18) out of whom 19 (65.5%) were on antiretroviral therapy. Five (17.2%) of the 29 participants were in the first clinical stage of the disease whereas 19 (65.5%) and 5 (17.2%) were in stages two and three, respectively. Low vision occurrence was lower in the ART group (9.8%) than in the non-ART group (11.1%). However, there was no significant association between low vision and ART status (*χ*^2^ = 0.24, *p* = 0.62).

Out of the 295 participants who took part in the study, 17 (5.8%) had HIV related ocular disorders of whom three were bilateral cases (making 20 eyes in all). Of these 17 participants, 4 (23.5%) were males and the remaining 13 (76.5%) were females. There was no significant association between manifestation of ocular disorders and sociodemographic factors such as gender (*p* = 1.00), age (*p* = 0.60), occupation (*p* = 0.38), and duration of infection from the time of diagnosis (*p* = 0.81). There was also no significant difference between the mean ages of patients who manifested ocular disorders and those who did not (*t* = 0.2, *p* = 0.80).

The most prevalent HIV related ocular disorder in this study was retinal microvasculopathy (10, 58.8%). The mean CD4 count for participants with this disorder was 142 cells/mm^3^ with a mean viral load of 20,308 copies/mL. Herpes zoster ophthalmicus and* Toxoplasma* retinochoroiditis each accounted for 2 (11.8%) of the ocular disorders found. Participants who had HZO recorded a mean CD4 count of 269 cells/mm^3^ and mean viral load of 38,090 copies/mL. Cytomegalovirus retinitis was responsible for only 1 (5.9%) case and it occurred in a participant with a CD4 count of 136 cells/mm^3^ and the highest viral load count of 1,474,676 copies/mL. Neuroophthalmic disorders found were Bell's (hemifacial) palsy and optic neuritis, each accounting for (1, 5.9%) of the cases.  Table 1 shows the various ocular disorders found in the patients. Mean viral load copies differed significantly between participants who manifested various ocular disorders (*F* = 362.7, *p* < 0.001) where CMV retinitis was associated with the highest viral load copies. However, the mean differences of CD4 counts did not differ significantly between the different ocular disorders (*F* = 2.5, *p* = 0.20).  Table 2 shows a comparison of mean CD4 counts/viral loads between various ocular disorders manifested.

Participants who had HIV related ocular disorders had significantly lower mean values of nadir CD4 counts (172 cells/mm^3^) when compared to those who did not have ocular disorders (309 cells/mm^3^) (*p* = 0.012). This mean CD4 count (172 cells/mm^3^) indicates that the patients who manifested HIV related ocular disorders had severe immune suppression (i.e., CD4 < 250 cells/mm^3^). Also, patients who manifested HIV related ocular disorders recorded significantly higher mean values of viral load copies as compared to their counterparts who did not manifest ocular disorders (*p* = 0.006). However, no significant association was found between ocular disorders and current CD4 counts (*p* = 0.10).  Table 3 compares the mean values of CD4 counts and viral loads between patients who manifested HIV related ocular disorders and those who did not.

Non-HIV related ocular conditions such as nonneurological blepharoptosis, pingueculae, pterygia, conjunctivitis, cataract, and glaucoma suspects were also found in the study population. Majority (11, 64.7%) of the participants who had HIV related ocular disorders were at stage two of the disease while 4 and 2 were at stages one and three, respectively. There was, however, no association between ocular manifestation and clinical stage of the disease (*χ*^2^ = 1.213, *p* = 0.724). Again, no association was found between ART usage and ocular manifestation (*χ*^2^ = 0.110, *p* = 1.000).

## 4. Discussion

Previous studies have always considered the associations between HIV related ocular manifestations and CD4 counts but not viral loads. The current study sought to evaluate the associations between presenting ocular disorders and CD4 counts/viral load copies in HIV positive patients attending the Cape Coast Teaching Hospital, Ghana. The 5.8% prevalence of HIV related ocular disorders in the present study was lower than in a recently published study from the Ashanti region of Ghana where the authors reported ocular complications in 48% [13]. That study included allergic conjunctivitis and cataract as complications resulting from HIV infection which may have accounted for the higher numbers. Our present finding is also lower than the 25.3% reported by Bekele et al. [8] in Jimma, Ethiopia, 12.3% in Northern Nigeria [14], 50% in the US [15], 60% in Morocco [16], and 52% in Brazil [17]. The fewer ocular lesions found in this study could also be attributed to the HIV disease status of our patients. For instance, majority of our participants (75.9%) were in stages one and two of the disease in which ocular manifestations are known to be fewer [18]. In the other study from Ghana, majority of the patients were in stage three of the disease [13]. Also, all the participants in our study who were supposed to be on ART as per the guidelines in Ghana [19] were on the therapy. ART usage is known to decrease the prevalence of HIV/AIDS-related ocular diseases [8]. It is understandable therefore that the prevalence in this study was relatively lower than it was in other studies that were done in the pre-ART era. The current study particularly showed that ocular manifestations in HIV infection are related to profound immune suppression and correspondingly high viral load copies. The fact that manifestation of ocular disorders was associated with nadir CD4 counts and not current CD4 counts implies that the ocular disorders occurred at the time of severe immune suppression.

The finding of 9.8% low vision in this study was higher than the 3.1% reported by Abokyi et al. [20] also in the Cape Coast Metropolis in Ghana. HIV retinal microvasculopathy being the most common HIV related ocular manifestation is consistent with the recent report from the Ashanti region, Ghana [13]. Our findings are also similar to those of Jabs [15] in USA, Assefa et al. [7] in Ethiopia, and Sahoo [21] in Tanzania where HIV related retinal microvasculopathy constituted 50%, 24%, and 25% of ocular complications, respectively. Jabs in his study found that the occurrence of HIV retinopathy was related to the stage of the disease [15]. The most common types of retinal microvasculopathy in this study were cotton wool spots in different retinal quadrants with or without retinal hemorrhages but their magnitude may be underestimated because they are classically transient and asymptomatic as is the case with other African countries [8, 22].

Herpes zoster ophthalmicus as the second most common ocular disorder was comparable with reported cases from Nigeria and Cameroon [22, 23]. Ayena et al. also reported herpes zoster ophthalmicus as the second commonest ocular manifestation of HIV infection in Togo [24]. Kehinde et al., however, reported herpes zoster ophthalmicus as the commonest ocular manifestation in patients with HIV/AIDS infection in Nigeria [14]. The disparities noted above may be due to the fact that the patients were at different stages of immune suppression in different population groups studied. The participants in our study and those in Togo were at different stages of treatment with ART, while those in the Nigerian study did not seem to be on therapy.

The finding on CMV retinitis is consistent with that in Ethiopia [25] and Tanzania [26] where CMV retinitis was responsible for 6.2 and 7.2%, respectively, of ocular complications in HIV/AIDS infection. Studies in Brazil [17] and Thailand [27], however, reported higher episodes of CMV retinitis of 25 and 33%, respectively, of ocular complications in HIV patients. CMV retinitis is reported to occur in patients with extreme immune suppression, usually with CD4 count of <50 cells/mm^3^ [9]. The very low prevalence of CMV retinitis seen in this study may not be a direct reflection of lower incidence but, as suggested by other researchers, of the notion that perhaps these patients die from other systemic opportunistic infections before their CD4 counts fall low enough to allow the development of CMV retinitis [28]. In the current study, only one patient had a CD4 count less than 100 cells/mm^3^ and majority, 69.5%, were on ART regimen. Spontaneous resolution of CMV retinitis has been reported in patients with increased CD4 count relating to antiretroviral therapy [29]. Other studies also found CMV retinitis to be rare among African patients [8, 29]. The recent publication from Ghana found no case of CMV retinitis [13]. These observations may explain the lower incidence of CMV retinitis in our study population and other African countries while Jabs et al. found 37% of CMV retinitis in the US even in the post-ART era [15]. The finding of 11.8% of neuroophthalmic disorders is similar to 9.6% by Assefa et al. in Ethiopia [7]. From Tanzania, Sahoo reported 6% of neuroophthalmic complications comprising papilloedema, optic atrophy, and cranial nerve palsies [21]. While optic neuritis is a common finding in HIV patients, Bell's palsy is not frequently reported. Seven cases of Bell's palsy in HIV patients have been reported in India [30].

## 5. Conclusion

Consistent with other findings, our study suggests that ocular manifestations in HIV patients are heightened by reduced CD4 counts and corresponding higher viral load copies. Particularly, CMV retinitis was associated with extremely high viral load copies though it was very rare.

## Figures and Tables

**Table 1:** Ocular disorders among participants.

Ocular disorder	*n* (%)
Cytomegalovirus retinitis	1 (5.9)
Herpes zoster ophthalmicus	2 (11.8)
HIV retinal microvasculopathy	10 (58.8)
*Toxoplasma* retinochoroiditis	2 (11.8)
Bell's (hemifacial) palsy	1 (5.9)
Optic neuritis	1 (5.9)
*Total*	*17 (100)*

**Table 2:** Comparing mean CD4 counts/viral loads between various ocular disorders manifested.

HIV biomarker	Mean count						*F*	*p* value
	CMV R	HZO	HIV RM	TR	ON	BP		
Nadir CD4 (cells/mm^3^)	136	269	142	342	278	148	2.5	0.20
Viral load (copies/mL)	1,474,676	38,090	20,308	146	18,060	41,120	362.7	<0.001

CMV R: cytomegalovirus retinitis; HZO: herpes zoster ophthalmicus; HIV RM: HIV retinal microvasculopathy; TR: *Toxoplasma* retinochoroiditis; ON: optic neuritis; BP: Bell's palsy.

**Table 3:** Comparing mean CD4 counts/viral loads between patients who manifested ocular disorders and those who did not.

HIV biomarker	Mean count (CI)		*t*	*p* value
	Participants with ocular disorders	Participants without ocular disorders		
Nadir CD4 count (cells/mm^3^)	172 (92.2–251.6)	309 (279.9–339.1)	2.5	0.012
Current CD4 count (cells/mm^3^)	660 (438.8–880.2)	807 (764.1–849.6)	1.7	0.10
Viral load (copies/mL)	165,270 (164883.1–495420.3)	31,992 (15919.2–48065.2)	2.8	0.006
